# Hepatitis C Virus: Insights Into Its History, Treatment, Challenges, and Future Directions

**DOI:** 10.7759/cureus.43924

**Published:** 2023-08-22

**Authors:** Luis A Bernal, Varun Soti

**Affiliations:** 1 Internal Medicine, Lake Erie College of Osteopathic Medicine, Elmira, USA; 2 Pharmacology and Therapeutics, Lake Erie College of Osteopathic Medicine, Elmira, USA

**Keywords:** hepatitis c virus, pharmacoeconomic burden, direct-acting antivirals, ribavirin, interferon alfa, hepatitis c virus (hcv)

## Abstract

Hepatitis C virus (HCV) is a global public health concern with significant impacts. It primarily spreads through blood-to-blood contact, such as sharing needles among drug users. Given the wide prevalence of risk factors, HCV continues to pose a major threat. Hence, it is crucial to understand its characteristics, structure, and genotypes to prevent, treat, and potentially eradicate it. This narrative review aims to explore the history of HCV treatment, highlight the breakthroughs achieved with direct-acting antiviral (DAA) therapy, address potential barriers to HCV eradication, and discuss future treatment possibilities. For this article, relevant studies were identified using various databases, including PubMed, ClinicalTrials.gov, and Journal Storage. The literature search revealed that after identifying HCV and studying its characteristics, interferon alfa and ribavirin became primary treatment options. However, due to their limited coverage against different HCV genotypes, ethnic variations, and suboptimal sustained virological response, the development of DAAs became essential. Combining various DAAs, such as sofosbuvir and velpatasvir, for a duration of 12 weeks has become the standard HCV treatment, with effectiveness against most genotypes. Additionally, ongoing clinical trials have shown promising results for other drugs such as CDI31244/sofosbuvir/velpatasvir, sofosbuvir/coblopasvir, and daclatasvir/asunaprevir. Despite the success of DAAs and ongoing efforts to discover more effective treatments, the high costs of DAAs pose a significant challenge to eradicating HCV, as not all patients can afford these expensive therapies. Furthermore, the ability of HCV to mutate limits the potential for vaccine development. Therefore, it is crucial to focus on developing more cost-effective strategies to control the spread of HCV and create novel, highly effective, and affordable DAAs.

## Introduction and background

Hepatitis C virus (HCV) is a significant global health concern due to its high prevalence and the substantial impact it has on public health. With an estimated global prevalence of 2.5%, HCV infections remain a substantial burden worldwide [1]. The virus is primarily transmitted through blood-to-blood contact, with common forms of transmission including receiving blood products, particularly transfusions, and sharing contaminated needles among intravenous drug users. Understanding the characteristics, structure, and genotypes of the virus is essential in developing effective strategies for prevention, treatment, and eradication [2].

HCV is an enveloped ribonucleic acid virus belonging to the *Flaviviridae* family. It exhibits high genetic variability, resulting in the classification of multiple genotypes and subtypes. The genomic variability of HCV poses challenges for developing a universal vaccine and long-term protection [3]. The virus primarily targets the liver, leading to chronic infection in a significant proportion of individuals. The hepatic effects of HCV infection can range from mild and acute inflammation to chronic effects, including cirrhosis (15-20%), liver failure (5-30%), and hepatocellular carcinoma (20%) [4].

The specific objective of this paper is to review the history of HCV treatment, from the early attempts using interferon (IFN)-alfa and ribavirin to the revolutionary breakthrough of direct-acting antiviral (DAA) therapy. We will discuss the challenges faced during the development of HCV treatments and the remarkable improvements achieved with the introduction of DAAs. Furthermore, we will explore the barriers to full eradication, such as the high cost of medications, limited availability of generics, and the need for a vaccine. Finally, we will examine the future of HCV treatments, including ongoing research and potential strategies to optimize treatment outcomes and reduce the global burden of HCV infection.

By addressing these goals and objectives, this review aims to contribute to the existing literature on HCV treatment and provide valuable insights into the advancements, challenges, and future directions in the field. Through a narrative review, we seek to enhance understanding and awareness of HCV, ultimately contributing to the efforts aimed at preventing, treating, and eradicating this significant public health issue.

## Review

Literature search and study selection

**Figure 1:**
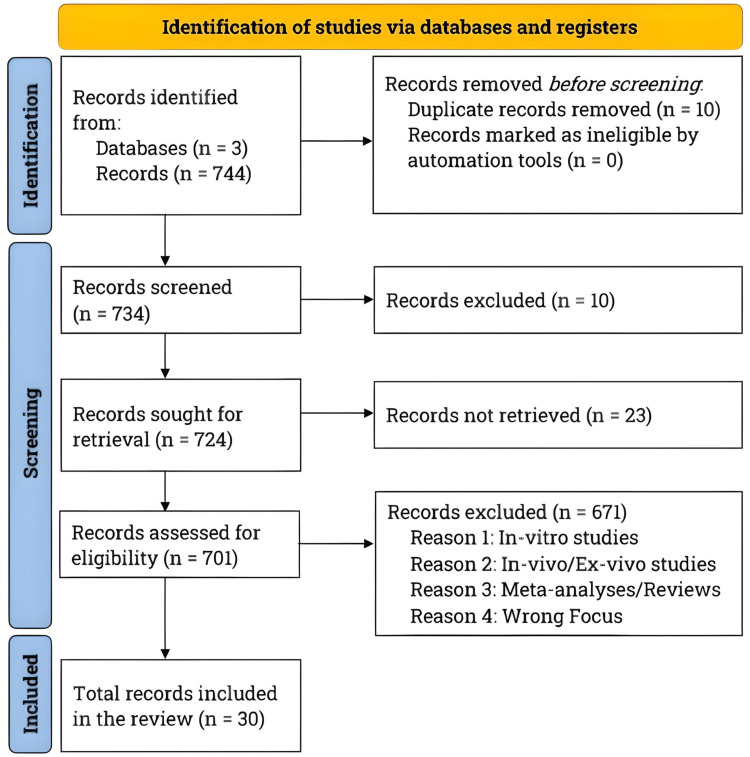
Literature search and study selection. We conducted a comprehensive literature search using PubMed, ClinicalTrials.gov, and Journal Storage. We specifically looked for articles written in English that focused on the evolution of the hepatitis C virus (HCV) initial drug treatment, breakthroughs in direct-acting antivirals, ongoing investigations of promising drugs, and studies examining the pharmacoeconomic burden. After carefully screening the studies, we narrowed our selection to 30, including seven studies that specifically explored outdated HCV treatments, current treatment regimens, and potential future options.

We conducted a comprehensive literature search from December 2022 to March 2023, strictly adhering to the Preferred Reporting Items for Systematic Reviews and Meta-Analyses (PRISMA) guidelines [5]. We utilized sources, including PubMed, ClinicalTrials.gov, and Journal Storage. Figure 1 illustrates the PRISMA flowchart, clearly depicting the study selection process and the total number of studies reviewed to document the advancements in HCV treatment.

To be included in this paper, the reviewed studies had to be clinical, published in English, and focused on HCV treatment, advancements, and future developments. We did not restrict our search to a particular period or decade. We reviewed a total of 30 studies, with seven specific studies focusing on the early HCV treatment, current treatment regimen, potential future treatment options, and barriers to eradicating HCV, and assigned a level of clinical evidence as per the previous literature [6].

Discovery and history of HCV

Hepatitis, a condition characterized by liver inflammation, has been a part of human history for ages. Many people, unfortunately, experience symptoms that include abdominal pain, tiredness, jaundice, and in severe cases, liver failure and death. Only in the 20th century did scientists discover that hepatitis primarily resulted from viruses infecting liver cells. Researchers subsequently classified viral hepatitis into two distinct diseases, both potentially serious but differing in transmission and impact on health. “Hepatitis A” spreads through person-to-person contact or contaminated food and water, causing an acute yet temporary illness with a short incubation period. “Hepatitis B,” on the other hand, transmits through blood and bodily fluids, resulting in a chronic infection with a more extended incubation period [7].

Given the rise of hepatitis cases associated with blood transfusions, it became crucial to identify the viruses, especially the blood-borne agent responsible for hepatitis B, to screen and prevent disease transmission via the blood supply. In 1963, researchers discovered a significant protein from the hepatitis B virus, which paved the way for blood testing. This protein was the serum antigen, a constituent of the hepatitis B virus, which exhibited a structure composed of 22-nanometer rods and spheres present in the bloodstream of infected individuals. It encompassed three envelope proteins of the hepatitis B virus, namely, L, S, and M, which are currently referred to collectively as hepatitis B surface antigens. Although implementing screening procedures and excluding infectious donors helped decrease post-transfusion hepatitis cases by 25-50%, scientists believed that the remaining cases were either the hepatitis A virus or the hepatitis B virus that bypassed the screening process [8].

However, in the mid-1970s, researchers made a significant breakthrough. They discovered the hepatitis A virus, collaborating with the National Institute of Health Clinical Center’s Division of Transfusion Medicine. It became evident that the remaining hepatitis cases were neither hepatitis A nor hepatitis B, indicating the presence of a distinct virus causing liver damage. This newfound disease could occur through infected blood, like hepatitis B. It could lead to chronic infection and liver cirrhosis, albeit with a higher likelihood of chronic illness among adults than hepatitis B. Interestingly, individuals affected by this disease hardly exhibited acute symptoms. The absence of overt signs made it possible for the disease to progress to a chronic stage undetected. Despite the passage of 15 years, the elusive cause of this condition remained a mystery, thus earning it the name “non-A, non-B hepatitis” [9].

In 1989, scientists at a California-based biotechnology company known as Chiron made a groundbreaking discovery in collaboration with investigators from the Centers for Disease Control and Prevention (CDC) [10]. They identified a new virus, officially named hepatitis C or HCV, previously known as non-A and non-B hepatitis. They accomplished this feat by creating a random-primed complementary deoxyribonucleic acid (cDNA) library from plasma taken from patients harboring the unidentified non-A, non-B hepatitis pathogen. They performed screening using serum from a patient diagnosed with non-A, non-B hepatitis infection. Consequently, they isolated a cDNA clone that encoded an antigen specifically associated with non-A, non-B hepatitis infections. Interestingly, this clone did not originate from host DNA but rather from a ribonucleic acid (RNA) molecule in non-A, non-B hepatitis infections. This positive-stranded RNA molecule, consisting of a minimum of 10,000 nucleotides, was found to code for the non-A, non-B hepatitis antigen. This remarkable medical advance paved the way for developing tests to detect HCV and screen blood donations. Over the following years, improvements in testing methods successfully eradicated HCV from the blood transfusion supply [11].

The identification of HCV also spurred further research and led to studies focused on determining the molecular structure of the virus, a critical step in designing drugs that could target specific components of the virus and hinder its replication. This vital information also led to more accurate diagnoses and a better understanding of the prevalence of HCV. In the Western world, HCV was the leading cause of hepatitis, liver cirrhosis, and liver carcinomas [12].

By adopting a collaborative approach, Chiron, in partnership with the CDC, made significant contributions in identifying and combating HCV. Their work enabled the development of diagnostic tools and effective screening measures and laid the foundation for targeted treatments. This groundbreaking research was crucial in improving HCV’s prognosis and overall management, offering hope for millions affected by this widespread and debilitating disease. However, developing effective treatments for HCV was fraught with medical and scientific challenges, as early treatments were often ineffective and had severe side effects [13].

First-generation treatments, challenges, and advancements

In the pre-HCV era, medical scientists relied on IFN-alpha for treating this mysterious chronic illness. IFN-alpha, naturally produced by immune cells in response to viral infections or stressors, was delivered through injections. It created an “interference” with virus replication, safeguarding cells from infection. Given its broad-spectrum antiviral properties, scientists logically explored IFN-alpha as a therapeutic tool against the unidentified virus causing non-A, non-B hepatitis [14].

In 1984, researchers initiated a pilot study of IFN by recruiting 10 patients at the National Institutes of Health (NIH) Clinical Center in Bethesda, Maryland. They administered patients daily doses of IFN-alpha for 16 weeks while closely monitoring their liver health through blood tests measuring liver damage markers. The trial yielded immediate and dramatic results, with most patients exhibiting improved liver health within a month of treatment. Despite relapses after discontinuing IFN-alpha treatment at four months, reintroducing the therapy enhanced liver health that remained normal even as the dosage gradually reduced and eventually discontinued after a full year. While some patients demonstrated minimal responses to IFN-alpha therapy and others experienced a response followed by relapse, half of the trial participants displayed no signs of liver infection during long-term follow-ups that extended 10 to 25 years. These fortunate individuals became the inaugural patients to be cured of the disease, now known as hepatitis C [15].

Despite the encouraging initial results, more extensive clinical trials dampened expectations regarding IFN-alpha. The studies showed significant patient outcomes variation, with single-agent IFN-alpha treatment generally yielding a low success rate regarding sustained virologic response (SVR). SVR refers to the absence of detectable virus for at least 24 weeks after treatment discontinuation, indicating a high likelihood of treatment success and prevention of relapse. Treatment with IFN-alpha alone typically resulted in less than 20% SVR rates. However, combination therapy incorporating IFN-alpha and other antiviral drugs showed promise [16]. One such drug, ribavirin, initially demonstrated moderate and temporary effects on viral levels when used as a stand-alone therapy [17]. Subsequent studies highlighted the superiority of the IFN-alpha and ribavirin combination compared to IFN-alpha alone, with SVR rates reaching 30% to 40% [18].

Another significant improvement occurred when scientists modified IFN-alpha chemically to extend its duration in the body. Termed “pegylated” IFN, combined with ribavirin, became the standard of care for hepatitis C patients, achieving SVR rates of 66% to 87% [19]. While IFN-based therapy demonstrated success in more than half of patients, it often coincided with adverse effects such as fever, fatigue, muscle aches, and depression, and these side effects frequently necessitated dose and duration limitations for treatments [20]. It underscored the need for further research and better therapeutic options. Nevertheless, these initial trials provided valuable insights into how HCV responds to or resists therapy and offered vital clues about its biology and resilience. This information served as a foundation for designing more effective treatments and heralded the emergence of DAA drugs. This breakthrough was a pivotal moment in the history of medical treatment of HCV [21].

DAA therapy as the mainstay treatment

A deeper understanding of the molecular components of HCV helped identify ideal targets for drug development. These significant breakthroughs included an enzyme called HCVpolymerase, which is crucial for the replication of the virus’s genetic material, a protease utilized by HCV to process its structural components prior to assembly, and proteins called HCV non-structural 5 (NS5) A and NS5B proteins, which play a critical role in virus replication and regulation of the cell’s response to IFN. This monumental discovery ushered in a new era of DAAs exclusively designed to target HCV directly [22].

The journey began in 2011 with the approval of the initial protease inhibitors by the United States Food and Drug Administration. These drugs, telaprevir and boceprevir, alongside subsequent similar drugs, specifically targeted the HCV protease, a key player in viral replication [23-25]. When combined with pegylated IFN and ribavirin, protease inhibitors yielded impressive SVR rates of up to 75%. However, this triple therapy also introduced additional side effects to those already present with peg-IFN and ribavirin. Regardless, the success of HCV-specific protease inhibitors demonstrated the virus’s vulnerabilities that researchers could exploit by developing well-designed and properly administered drugs [26].

Scientists developed and rigorously tested several new anti-HCV drugs in the following years. These included ombitasvir, ledipasvir, daclatasvir, elbasvir, and velpatasvir, which targeted and blocked the HCV NS5A protein, while sofosbuvir and dasabuvir inhibited the HCV NS5B protein. Initially, researchers conducted trials with the combination of these drugs alongside pegylated IFN and ribavirin or another protease inhibitor, resulting in SVR rates of at least 80% [27,28].

The most notable breakthrough came in 2015 when clinical trial results unveiled that the combination of two new DAAs, sofosbuvir and velpatasvir, achieved an impressive SVR of 99% across all known genotypes of hepatitis C. This combination exhibited better efficacy than previous medications, eliminating the need for IFN-alfa, ribavirin, or telaprevir, thereby reducing the likelihood of severe side effects and non-compliance-related relapse. This new drug regimen also introduced an oral formulation, eliminating the need for injections. Moreover, sofosbuvir/velpatasvir significantly shortened the treatment duration to 12 weeks from the previous 48 weeks [29].

Another highly successful and potentially more effective DAA combination was sofosbuvir and ledipasvir. The administration of these two DAAs resulted in SVR rates reaching 95-99%. Remarkably, this combination achieved such results with treatment durations of only eight to 12 weeks. After years of dedicated research, a genuine cure for hepatitis C emerged, proving effective for almost everyone [28].

Barriers to full eradication of HCV

The expeditious development of highly efficacious drugs to treat HCV is more than just life-saving. With an estimated global prevalence of 2.5% [1], millions of undiagnosed cases could be due to HCV's chronic nature and lack of acute symptoms. Genotype 1, equivalent to almost half of HCV infections worldwide, is predominant in East Asia [30,31]. Although, the most frequently reported risk factor for HCV infections is intravenous drug use, with almost 39% prevalence among current abusers and responsible for 8% of worldwide HCV infections [32].

Despite newer DAAs against HCV making treatment for active drug users possible, there is still skepticism regarding the World Health Organization's 2030 goal of eradicating HCV [32]. One major challenge has been the genomic variability of HCV outer proteins and their ability to mutate frequently, rendering almost no residual protection from future infections [33]. Treatment for HCV was previously based on a numbers game, as mono treatment could lead to resistance and more mutation potential, similar to tuberculosis or human immunodeficiency virus infections. Vaccine development for HCV has faced significant obstacles, and its feasibility is uncertain [34].

However, to achieve global eradication of HCV infection, the healthcare community must surmount more obstacles in addition to acknowledging the lack of a vaccine and reluctance to treat drug users. The cost of the branded drugs and the unavailability of generics pose significant barriers to providing patients with necessary treatment. Currently, in the United States, three-month treatment courses for available DAAs are prohibitively expensive, costing as much as $94,500 to $150,000 per treatment [35]. Such costs make curing HCV impossible for many, including those without medical insurance, driving some to turn to other countries or illegal means for their medications. Seeking affordable treatment can be costly and inaccessible to many patients. The pharmacoeconomic burden of an HCV infection is extensive and can have detrimental effects on society. Furthermore, reducing this burden by 10 to 20 years after treatment undeniably highlights the importance of pursuing global HCV eradication. This mission serves both humanitarian and economic purposes, making it a crucial endeavor [36].

HCV’s future treatment options

Imposing competition could be a feasible option to reduce the cost of DAA treatment for HCV. Despite the advent of DAAs, the innovation and amalgamation of existing drugs with new drugs have continued. For instance, in China, sofosbuvir, in combination with seraprevir (currently in phase III clinical trials), has been observed to attain an SVR of 98% among Chinese individuals who were treated unsuccessfully with multiple formulations of pegylated IFN-alfa [37]. Another new drug, coblopasvir, has been tested with sofosbuvir, achieving an SVR of 97% among patients with different HCV genotypes [38]. In phase III clinical trials, asunaprevir tested with daclatasvir recorded an SVR of 92% [39].

Besides discovering newer drugs, other treatment modalities have been thoroughly studied. The standard treatment length recommended for currently available treatments for HCV in the United States is a 12-week course of dual direct antiviral therapy, following current guidelines. However, some studies have aimed to curtail the course duration while upholding a high SVR among participants. In a small clinical trial, a novel non-nucleoside reverse transcriptase inhibitor was combined with sofosbuvir/velpatasvir to treat treatment-naïve genome in one patient infected with HCV, using a shortened trial of only six weeks. The trial reported an SVR of 75% amongst the trial participants, thus marking it as a positive step in the researchers' efforts to ameliorate HCV treatment while reducing costs [40]. Refer to Table 1 for the evolution of drugs for treating HCV.

**Table 1:** Hepatitis C virus treatment evolution. This table provides an overview of the progression in drug treatment for the hepatitis C virus, depicting its development from the early stages to the current treatment regimen and future potential treatments. It showcases the remarkable evolution in managing this virus, from past breakthroughs to present advancements, and offers insights into the future. SVR: sustained virological response.

Authors	Study design	Level of clinical evidence	Sample size	Drug(s)	SVR
Antiquated treatment(s)					
Abonyi and Lakatos (2005) [19]	Non-randomized	II	650	Pegylated interferon alfa/ribavirin	66-87%
Current treatment(s)					
Sulkowski et al. (2021) [28]	Randomized	I	1,128	Ledipasvir/sofosbuvir (arm 1), elbasvir/grazoprevir (arm 2)	95-99% (arm 1), 97% (arm 2)
Feld et al. (2015) [29]	Randomized with placebo	I	624	Sofosbuvir/velpatasvir	99%
Treatment(s) under development					
Kong et al. (2021) [37]	Non-randomized	II	205	Seraprevir/sofosbuvir	98%
Gao et al. (2020) [38]	Non-randomized	II	371	Sofosbuvir/coblopasvir	97%
Wei et al. (2018) [39]	Randomized with placebo	I	155	Daclatasvir/asunaprevir	92%
Chua et al. (2021) [40]	Non-randomized	II	12	CDI31244/sofosbuvir/velpatasvir (shortened six-week trial)	67%

Various studies have shown that certain areas need improvement to optimize treatment delivery. For instance, treatment adherence strategies are crucial in achieving SVR, but they are usually more critical in the first eight weeks of treatment and lose significance in the last four weeks. Moreover, switching between on and off days of DAA intake was not associated with decreased SVR after any month of treatment. These findings indicate that dosage adjustment could hold the key to better treatment outcomes in the future [41].

## Conclusions

The discovery of HCV and its rapid differentiation from hepatitis A and B viruses paved the way for screening and therapeutic developments to combat HCV. Initial treatments, such as IFN-alfa and ribavirin, provided infected individuals with an opportunity for SVR, albeit with imperfections. With continuous research and advancements, scientists swiftly developed DAAs. DAA therapies have proven highly effective and have become the cornerstone of HCV treatment. However, the global mission to eradicate HCV faces several challenges, including the high pricing of DAAs and the absence of a vaccine. Effectively managing HCV remains challenging due to its widespread prevalence and associated risk factors. Nevertheless, significant progress has been made in reducing mortality, effectively treating, and improving the overall well-being of HCV patients.
